# Developing a complex intervention targeting antipsychotic prescribing to nursing home residents with dementia

**DOI:** 10.12688/hrbopenres.13228.1

**Published:** 2021-02-19

**Authors:** Kieran A. Walsh, Stephen Byrne, Jenny McSharry, John Browne, Kate Irving, Eimir Hurley, Helen Rochford-Brennan, Carmel Geoghegan, Justin Presseau, Suzanne Timmons

**Affiliations:** 1 Centre for Gerontology and Rehabilitation, School of Medicine, University College Cork, Cork, T12XH60, Ireland; 2 Pharmaceutical Care Research Group, School of Pharmacy, University College Cork, Cork, T12YN60, Ireland; 3 School of Public Health, University College Cork, Cork, T12K8AF, Ireland; 4 Health Behaviour Change Research Group, School of Psychology, National University of Ireland, Galway, Galway, H91TK33, Ireland; 5 School of Nursing, Psychotherapy and Community Health, Dublin City University, Dublin, Ireland; 6 Centre for Health Policy and Management, Trinity College Dublin, Dublin, Ireland; 7 Expert by Experience, N/A, N/A, Ireland; 8 Irish Dementia Working Group, The Alzheimer Society of Ireland, Dublin, Ireland; 9 Ottawa Hospital Research Institute, The Ottawa Hospital, Ottawa, K1H 8L6, Canada; 10 School of Epidemiology and Public Health, University of Ottawa, Ottawa, K1G 5Z3, Canada

**Keywords:** Intervention development, complex interventions, MRC framework, behaviour change, dementia, antipsychotics, nursing homes, patient and public involvement, GUIDED

## Abstract

**Background**: Antipsychotics are commonly prescribed to people living with dementia in nursing home settings, despite strong guideline recommendations against their use except in limited circumstances. We aimed to transparently describe the development process for a complex intervention targeting appropriate requesting and prescribing of antipsychotics to nursing home residents with dementia in Ireland, by nurses and general practitioners (GPs) respectively.

**Methods**: We report the development process for the ‘Rationalising Antipsychotic Prescribing in Dementia’ (RAPID) complex intervention, in accordance with the ‘Guidance for reporting intervention development studies in health research’ (GUIDED) checklist.  The UK Medical Research Council framework for developing and evaluating complex interventions guided our overall approach, incorporating evidence and theory into the intervention development process. To unpack the intervention development process in greater detail, we followed the Behaviour Change Wheel approach. Guided by our stakeholders, we conducted three sequential studies (systematic review and qualitative evidence synthesis, primary qualitative study and expert consensus study), to inform the intervention development.

**Results**: The RAPID complex intervention was developed in collaboration with a broad range of stakeholders, including people living with dementia and family carers, between 2015 and 2017. The finalised RAPID complex intervention was comprised of the following three components; 1) Education and training sessions with nursing home staff; 2) Academic detailing with GPs; 3) Introduction of an assessment tool to the nursing home.

**Conclusions**: This paper describes the steps used by the researchers to develop a complex intervention targeting antipsychotic prescribing to nursing home residents with dementia in Ireland, according to the GUIDED checklist. We found that the GUIDED checklist provided a useful way of reporting all elements in a cohesive manner and complemented the other tools and frameworks used. Transparency in the intervention development processes can help in the translation of evidence into practice.

## Introduction

Antipsychotics are a class of medication that are principally used for the treatment of schizophrenia and other psychotic disorders, including bipolar affective disorder ^1^. However, they are frequently used in people living with dementia, especially in nursing home settings, to treat the so-called ‘behavioural and psychological symptoms of dementia’ (BPSD) ^2, 3^, despite modest evidence of effectiveness and significant evidence of harms ^4– 6^. BPSD includes a range of behaviours (such as agitation, aggression, repetitive questioning and wandering) and psychological symptoms (such as depression, anxiety, apathy, psychosis and insomnia) that commonly occur in people living with dementia ^7^. The causes of BPSD are complex and poorly understood, but are thought to include disease-related factors, care giving factors, unmet needs in the person living with dementia, and environmental triggers. Given the complexity of causes of BPSD, there is no “one size fits all solution,” and approaches tailored to the person living with dementia, that always consider non-pharmacological interventions, are important ^7^.

Guidelines across jurisdictions strongly recommend against the first line use of antipsychotics for the management of BPSD, except when there is an imminent risk of harm to the person and/or to others, or when the person living with dementia is severely distressed by the symptoms ^8– 10^. Instead, guidelines consistently recommend the first line use of non-pharmacological interventions, using an individualised and person-centred approach at all times ^8– 10^. Though many different interventions conducted over the years have resulted in reduced inappropriate antipsychotic prescribing in nursing homes, there is limited evidence of long term effectiveness, with prescribing rates often returning to pre-intervention levels ^11^. Furthermore, while some reductions in antipsychotic prescribing to people living with dementia have been observed in recent years at a country level ^12– 14^, there is evidence emerging that the coronavirus disease 2019 (COVID-19) pandemic, which disproportionately affects nursing home residents with dementia, is associated with a significant rise in antipsychotic prescribing ^15, 16^.

Here, we describe the steps used by the research team to develop a complex intervention targeting antipsychotic prescribing to nursing home residents with dementia in Ireland. The aim was to develop an intervention that was evidence- and theory-based, involved people living with dementia and family carers in its development, and could potentially be sustained in practice. The purpose of the current paper is to transparently describe the development of this complex intervention, to enable replication, scale and spread if the intervention is shown to be effective, and also to facilitate learning on intervention development practice ^17^.

## Methods

### Background to the RAPID complex intervention

The ‘Rationalising Antipsychotic Prescribing in Dementia’ (RAPID) complex intervention was developed as part of the lead author’s doctoral studies (KW). The project was a collaboration between health and social care professionals, people living with dementia, family carers, advocacy groups and academics. Ethics approval for this project was granted by the Clinical Research Ethics Committee of the Cork Teaching Hospitals [ECM 4 (e) 13/10/15, ECM 3 (rrr) 21/06/16, ECM 3 (jjjjj) 09/08/16, ECM 4 (x) 19/01/16, ECM 3 (qqq) 21/06/16, ECM 3 (kkkkk) 09/08/16, and ECM 3 (kk) 10/01/17].

### Reporting of the intervention development process

The RAPID complex intervention was shaped by evidence and theory ^18– 20^, and developed using a mixed-methods research programme. Three sequential studies were used ^21– 23^, though in reality the process was non-linear, while decision-making was influenced by many different factors, such as stakeholder buy-in, data access and logistics. In this paper we describe the development process in accordance with to the ‘Guidance for reporting intervention development studies in health research’ (GUIDED) checklist ( Table 1) ^17^. The sections below describe the process of intervention development in relation to the 14 items of the GUIDED checklist ( Table 1). While other frameworks and tools are referred to throughout this paper, we use GUIDED as the main framework to report the intervention development process.

**Table 1.** GUIDED – a guideline for reporting intervention development studies *. Reproduced from Duncan *et al.* ^17^ under the terms of the Creative Commons Attribution 4.0 International (CC BY 4.0) license.

Item description
1. Report the context for which the intervention was developed.
2. Report the purpose of the intervention development process.
3. Report the target population for the intervention development process.
4. Report how any published intervention development approach contributed to the development process
5. Report how evidence from different sources informed the intervention development process.
6. Report how/if published theory informed the intervention development process.
7. Report any use of components from an existing intervention in the current intervention development process.
8. Report any guiding principles, people or factors that were prioritised when making decisions during the intervention development process.
10. Report how the intervention changed in content and format from the start of the intervention development process.
11. Report any changes to interventions required or likely to be required for subgroups.
12. Report important uncertainties at the end of the intervention development process.
13. Follow TIDieR guidance when describing the developed intervention.
14. Report the intervention development process in an open access format.

*Note that the full definitions for each item are available in Extended Data Table 1 ^28^

Each of these frameworks and tools serve different purposes; the UK Medical Research Council framework aims to provide a general framework for developing and evaluating complex interventions ^18^; the Behaviour Change Wheel describes a specific approach, based on evidence and theory, for developing behaviour change interventions ^19^; the Theoretical Domains Framework aims to identify and describe the factors that influence a behaviour ^20^; the Template for Intervention Description and Replication (TIDieR) checklist aims to improve the completeness of reporting and the replicability of interventions ^24^; the Context and Implementation of Complex Interventions (CICI) framework aims to simplify and structure intervention complexity in order to advance our understanding of whether and how interventions works ^25^; the Effective Practice and Organisation of Care (EPOC) taxonomy provides a classification system for describing and organising health systems interventions ^26^; and the Guidance for Reporting Involvement of Patients and the Public 2 – Short Form (GRIPP2-SF) checklist aims to improve the quality, transparency, and consistency of the patient and public involvement (PPI) evidence base ^27^.

The UK Medical Research Council framework and Behaviour Change Wheel approach guided our intervention development process from start to finish, while the Theoretical Domains Framework was used specifically during our primary qualitative study to identify the behavioural determinants of appropriate requesting and prescribing of antipsychotics to nursing home residents with dementia ^22^. The remaining frameworks and tools (i.e. TIDieR, EPOC, CICI and GRIPP2-SF) are used for the first time in the current study.

## Results

### 1. The context

Here, we use the CICI framework, and specifically the seven context domains (geographical, epidemiological, socio-cultural, socio-economic, ethical, legal and political) to describe the context in which the intervention was developed ^25^.

The RAPID complex intervention was developed over a two year period, from 2015 to 2017 ^25^. The intervention was developed in Ireland, with the aim of initial feasibility testing in an urban region in the south-west of the country. Though there is limited pharmaco-epidemiological data on the prevalence of antipsychotic prescribing in Ireland, international evidence indicates that approximately 30–40% of nursing home residents with dementia are prescribed an antipsychotic ^2^. In relation to socio-cultural issues, there is stigma surrounding BPSD and more broadly around dementia ^29^, and this has implications on how people living with dementia are treated and viewed by society. While non-pharmacological interventions are considered the gold standard for managing BPSD, there are resource constraints within nursing homes which can act as a barrier to their effective implementation ^21^. There are important ethical and legal issues surrounding antipsychotic prescribing to residents with dementia. Personal autonomy of the resident needs to be respected, however the safety of the resident and others needs to be considered, and this can be challenging in the context of BPSD ^22^. The nursing home regulator in Ireland, the Health Information and Quality Authority (HIQA) mandates the reporting of chemical and physical restraint usage in nursing homes ^30^, though this presents its own challenges as reporting guidance is considered to be non-specific ^22^, and any instance of restraint usage is self-reported by the nursing home ^22^. There has been a recent impetus for change in the system, with the launch of the Irish National Dementia Strategy in 2014 which identified inappropriate antipsychotic prescribing as a priority for action ^31^. A recommendation from the National Dementia Strategy was to develop a National Clinical Guideline on this topic that was specific for the Irish healthcare system, and this was completed in 2019 ^8^.

### 2. The purpose of the intervention development process

The overall aim of the intervention was to improve the appropriateness of requesting and prescribing of antipsychotics to nursing home residents with dementia, by nurses and GPs respectively. The aim of this development phase of the project was to develop an evidence- and theory-based complex intervention that could be delivered locally to staff providing care to residents with dementia, and that had the potential to be sustained in practice and scaled up across a large number of nursing homes.

### 3. The target population

The intervention is primarily targeted at nurses and GPs who provide care to nursing home residents with dementia. This is based on our previous qualitative findings which found that these two key stakeholder groups are the most central to the decision to request or prescribe an antipsychotic to a resident with dementia ^21, 22^. While the main target within the nursing home setting were nurses given their role in medication management, we considered that involving others that work in nursing homes (such as healthcare assistants) may be an important way of restructuring the social environment and promoting behaviour change ^19^. Hence the intervention involved nursing home staff (which comprised both nurses and healthcare assistants) along with GPs. It was envisaged that the intervention would ultimately benefit nursing home residents with dementia by reducing the inappropriate prescribing of antipsychotics to this population and thereby potentially reducing the risk of sedation, stroke, hospitalisation and mortality and potentially improving residents’ quality of life. This intervention also had the potential to reduce health and social care costs by potentially reducing the number of hospital admissions relating to inappropriate antipsychotic prescribing ^32^.

### 4. Contribution of published intervention development work to the process

**Figure 1.**
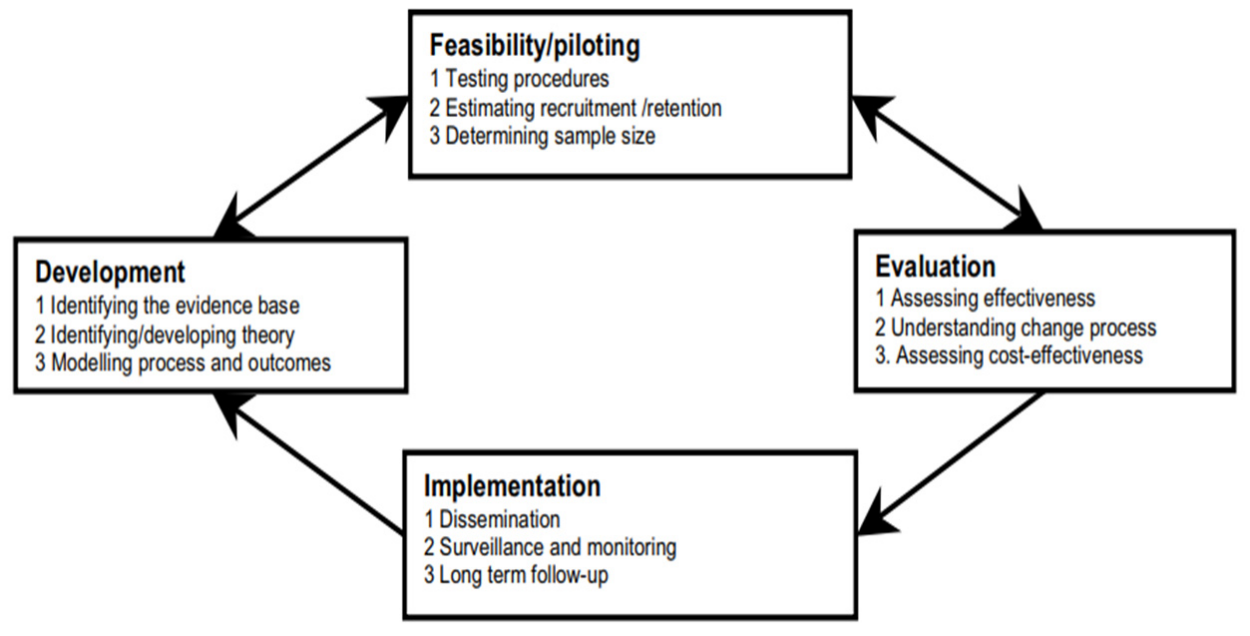
The UK Medical Research Council framework for developing and evaluating complex intervention ^[Bibr ref-18]^. "Reproduced from [Developing and evaluating complex interventions: the new Medical Research Council guidance, Craig Peter, Dieppe Paul, Macintyre Sally, Michie Susan, Nazareth Irwin, Petticrew Mark *et al*., 337, a1655, 2008] with permission from BMJ Publishing Group Ltd."

**Figure 2.**
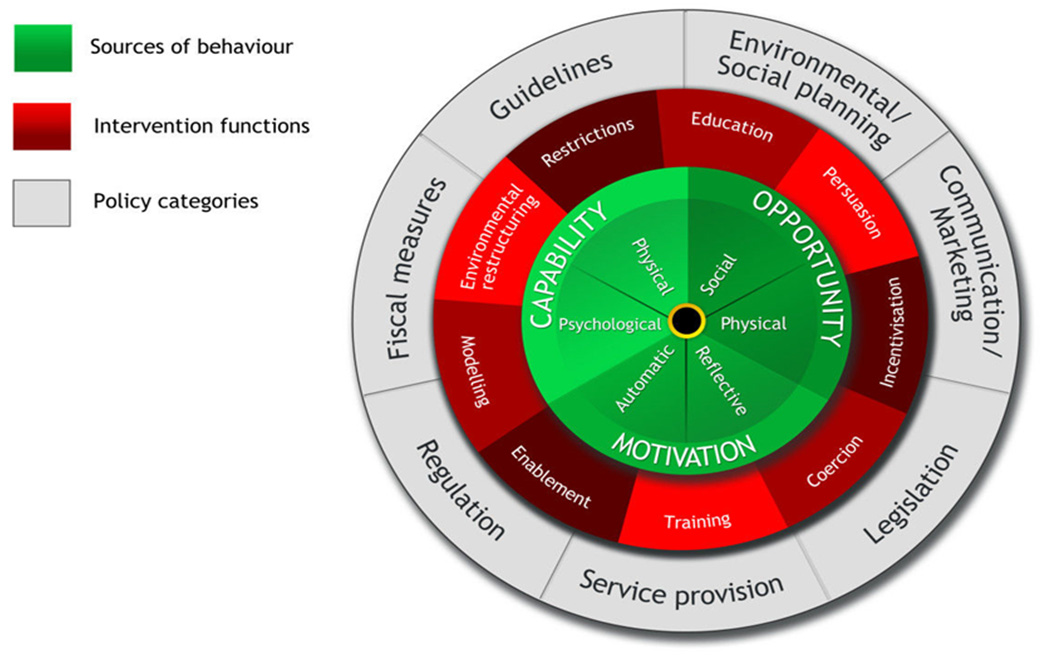
The Behaviour Change Wheel. Reproduced from Michie, van Stralen and West ^19^ under the terms of the Creative Commons Attribution 2.0 Generic (CC BY 2.0) license.

The UK Medical Research Council framework for developing and evaluating complex interventions guided our overall approach to this research ( Figure 1), incorporating evidence and theory into the intervention development process ^18^. Within the Development Phase of the UK Medical Research Council framework, we used the Behaviour Change Wheel guidance to develop intervention content, with a particular focus on identifying behaviour change techniques (BCTs) for the final intervention, by linking the behavioural determinants to potential intervention functions ( Figure 2) ^19, 33^. The use of the Behaviour Change Wheel approach in the development of the RAPID complex intervention is detailed in our previous publication ^23^.

### 5. How evidence from different sources informed the intervention development process

In developing our complex intervention, we based our decisions on findings from our previous research ^21– 23^, the effectiveness of interventions to reduce inappropriate antipsychotic prescribing ^11^, the advice of our advisory groups, the suitability of various theoretical approaches used in implementation science ^34^. and published evidence on the effectiveness of various implementation strategies ^35– 39^.

A systematic review published in 2014 by Thompson Coon *et al.* provided a comprehensive evidence-base for the effectiveness of different interventions to reduce inappropriate antipsychotic prescribing to nursing home residents with dementia ^11^. The review authors concluded that interventions to reduce inappropriate prescribing of antipsychotics to nursing home residents with dementia may be effective in the short term, but longer and more robust studies are needed. The review authors added that, “in order for prescribing levels to be reduced in the long term, the culture and nature of nursing home settings and the availability and feasibility of non-drug alternatives needs to be addressed” ^11^. The findings from this review encouraged us to address some of the research questions identified by the review authors. Therefore, we conducted three sequential studies which directly informed the intervention development process ^21– 23^. The first study was a systematic review and qualitative evidence synthesis which aimed to synthesise the findings from individual qualitative studies on decision-making and prescribing behaviours for antipsychotics in nursing home residents with dementia ^21^. The second study was a primary qualitative study which aimed to explore and interpret the determinants of appropriate prescribing behaviours among a range of individuals involved in the care of nursing home residents with dementia ^22^. The third study was a mixed methods expert consensus study which aimed to identify BCTs for inclusion in a complex intervention targeting antipsychotic prescribing behaviours ^23^.

Stakeholder involvement was also used throughout to inform the intervention development process. Advisory groups which were formed as part of this study comprising people living with dementia, family carers of people with dementia and healthcare professionals who provide care to nursing home residents with dementia, provided input into the intervention development process throughout the project (see item 9). Hence, the RAPID complex intervention was developed using a mixed-methods approach, using a broad range of stakeholders.

### 6. Use of existing published theory

The second sequential study we conducted was a primary qualitative study with healthcare workers and family members involved in the care of residents with dementia ^22^. This study used the Theoretical Domains Framework, an integrative framework of 14 domains of influences on behaviour developed by synthesising multiple behaviour change theories ^20^, to explore the determinants of the target behaviours for this complex intervention. Following Behaviour Change Wheel guidance, we linked the predominant Theoretical Domains Framework domains identified from this study to five intervention functions (Education, Persuasion, Training, Environmental restructuring, and Modelling) and 16 BCTs (see Item 13 below), and this process is outlined in detail in our previous publication ^23^.

According to the Behaviour Change Wheel, the final step of the intervention development process involves identifying the mode of delivery ^19^. This step requires translating the selected BCTs into a tangible intervention, aimed at our target behaviours, population group and setting, while considering broader implementation issues. Based on all available sources of evidence, along with discussions with our advisory groups, we considered the following four implementation strategies to be of particular importance to the RAPID complex intervention:

1. academic detailing (also called educational outreach, which is an approach aimed at improving prescribing practices using proactive outreach with non-commercial, evidence-based medical information in a user friendly format) ^39^
2. local opinion leaders (opinion leaders are people who are seen as likeable, trustworthy and influential, and may be able to persuade others to change their behaviour) ^35^.
3. multidisciplinary education meetings/workshops ^36^
4. printed educational materials ^37^.

In terms of the implementation and potential sustainability of the intervention in the nursing home setting where there is often rapid turnover of staff, we adopted the Diffusion of Innovations Theory ^40^. We theorised that by training local opinion leaders as ‘dementia champions’ and by equipping them with adequate tools and knowledge, they could act as early adopters within the setting and actively promote behaviour change among their colleagues. These ‘dementia champions’ could in principle implement the intervention throughout the setting, providing ongoing training to others who may not have attended the training, offsetting the high levels of staff turnover, and potentially embedding the behaviour change in practice.

### 7. Use of components from an existing intervention

Recently developed educational material that was considered to meet our study objectives formed the basis of the education and training delivered to nursing home staff as part of the RAPID intervention (developed by content experts from Dublin City University) and our academic detailing (developed by https://cep.health/ and https://deprescribing.org/). These materials were adapted for the purpose of our intervention and target audience with permission from the developers. Specifically, the education and training session delivered to nursing home staff was comprised of three previously developed topics (i.e. understanding and responding to the person with dementia, everyday ethics, and understanding emotion), while a new module was developed by the lead author (KW) in conjunction with the senior author (ST) (i.e. antipsychotic drug use in dementia). In total four topics were delivered to nursing home staff. With regards to the academic detailing with GPs, both tools were used in their entirety and were not modified. The RAPID assessment tool was developed *de novo* by the lead author (KW) in conjunction with the primary supervisor (ST) based on a rapid literature review of BPSD management checklists and guidelines.

### 8. Guiding principles

Our main focus was to design an intervention for sustainability and suitability within the Irish nursing home context, that balanced quality and safety, and that could potentially be continued by the nursing home after we had completed our study. To help us select the most appropriate intervention functions, we used the APEASE criteria (affordability, practicability, effectiveness, acceptability, side effects, and equity) outlined within Behaviour Change Wheel Guidance ^19^, and considered sustainability issues of different intervention options, as discussed in our previous study ^23^. Any developed intervention also needed to be feasible within our own resource constraints.

Our approach was guided by both the views of our PPI advisory groups of people living with dementia and family carers, as well as the views of our professional stakeholder group who provided care for nursing home residents with dementia (see item 9 below). We aimed to be as accommodating as possible to all perspectives on this topic, in so far as possible. Throughout the entire process, we were mindful of the aims of the Irish National Dementia Strategy, which seeks to progress the dual and overarching principles of personhood and citizenship by enabling people with dementia to maintain their identity, resilience and dignity ^31^.

### 9. Stakeholder involvement

The research team comprised individuals with a broad range of relevant experience, including pharmacy, geriatric medicine, nursing, health services research, academic detailing, dementia education, intervention development, behaviour change and implementation science. PPI was a central component of this project, whereby one PPI advisory group of people living with dementia and one PPI advisory group of family carers, who are experts by experience, provided advice to the research team throughout all stages of the project. In brief, PPI advisory group eligibility criteria included having an interest in research aimed at improving the quality of medication usage in nursing homes, and either being a person with dementia affiliated with the Irish Dementia Working Group (IDWG) of the Alzheimer Society of Ireland (ASI), or being a family member of any nursing home resident with dementia. The IDWG are a group of people living with dementia who advocate for better services, supports and policies for people living with dementia in Ireland ^41^. Recruitment of people living with dementia, was facilitated by the ASI, who allowed the lead author (KW) to present the planned project at a meeting of the IDWG. Recruitment of family members occurred at meetings and conferences involving family carers of people living with dementia, where the lead author (KW) discussed the planned project with attendees. Written informed consent was obtained from all PPI advisory group members. The PPI advisory group meetings of people living with dementia were co-facilitated by the Alzheimer Society of Ireland ^23^. The PPI advisory group meetings with family members were less structured, and although several face to face meetings took place, the majority of the interactions were via phone, email or letters. The key role played by the two PPI advisory groups throughout the development of this complex intervention, is outlined using the GRIPP2-SF checklist ^27^. (Extended Data Table 2) ^28^.

Alongside our PPI advisory groups, we separately consulted with professional stakeholders who were involved in providing care to nursing home residents with dementia (including general practitioners (GPs), psychiatrists of old age, nurses, pharmacists and geriatricians). These stakeholders were professionally known to the research team, and agreed to become advisors for the project. These individuals were consulted at various times throughout the project to gain an insight into the practicalities of providing care to nursing home residents with dementia. The consultations with the professional stakeholders tended to be less structured than that of the PPI advisory groups and occurred throughout the intervention development process.

The consultation process with all stakeholders is outlined in detail in our previous publication ^23^.

### 10. Changes in intervention content and format

Due to the iterative and formative nature of the intervention development process, there were some changes to the intervention content and format from the start of the intervention development process.

The most substantial change was in relation to the role of family members in the intervention under development. The PPI advisory groups strongly favoured a central role for family members in the intervention, as a means of advocating on behalf of the resident. Concerns emerged at an early stage, from the professional stakeholder groups regarding centrally involving family members in the intervention. Although we believed the involvement of family members to be important, several professional stakeholders cited past negative experiences with central family member involvement in antipsychotic decision-making as a reason to be cautious. When we initially attempted to recruit nursing homes, there was a reluctance to sign up, and this appeared to be due to their unease regarding centrally involving family members in the proposed intervention. Hence, despite our desire to adhere to our PPI advisory group members’ advice to centrally involve family members in the planned intervention, we took the decision to remove this aspect from the intervention in order to attain buy-in from prospective nursing home sites. Importantly, the critical role that family members play in advocating for their loved ones with dementia was not prevented by the intervention. In fact, nursing home staff were actively encouraged to involve family members when using the RAPID assessment tool that was developed as part of this study ( Box 1). Additionally, it was ethically right to inform family members of the ongoing intervention, to provide them with adequate information about the study, and to allow them to contact the research team should they have any queries about the study.

**Box 1. BCT Composition of RAPID Complex Intervention**

BCT – behaviour change technique

The procedures involved in the RAPID complex intervention are as follows (the 16 relevant BCTs are italicised and underlined in brackets):

•    The five intervention functions directed at nursing home staff will include: **Education, Training, Persuasion, Environmental Restructuring and Modelling**.

◦ During education and training session, nursing home staff will be provided with written and oral information regarding the risks and benefits of antipsychotics *( 5.1 Information about health consequences)* from experienced pharmacists, physicians and nurses *( 9.1 Credible source).* After presenting the evidence, staff will be asked to consider antipsychotics as the last resort when dealing with the majority of behavioural symptoms, rather than the first-line treatment *( 13.2 Framing/re-framing)* and will be encouraged to use non-drug alternatives instead of requesting antipsychotics in these instances *( 8.2 Behaviour substitution).* Through group discussions, staff members will share with each other, occasions where non-drug strategies worked and antipsychotics were not needed *( 15.3 Focus on past success).*
◦ At the same education and training session, nursing home staff will be introduced to the newly developed RAPID assessment tool which has the aim of aiding staff with the assessment of behavioural symptoms and ultimately reduce inappropriate requests for antipsychotics. Staff will be directed how to complete the RAPID tool via demonstration *( 6.1 demonstration of behaviour)* and also through written instructions accompanying the tool *( 4.1 Instruction on how to perform a behaviour).* The RAPID tool will focus staff’s attention on identifying and exploring patterns of events and triggers that occur in residents (e.g. repetitive actions, sun-downing, pain) *( 4.2 Information about antecedents)* that may ultimately lead to an inappropriate request for an antipsychotic, and to develop non-drug strategies to use in these situations to address these factors *( 1.2 Problem solving).* Staff will be encouraged to outline a detailed plan of how and when non-drug and/or drug interventions will be utilised in such situations *( 1.4 Action Planning, 1.2 Problem solving*). Staff will practice using the RAPID tool based on case studies provided in the education and training session *( 8.1 Behavioural practice/rehearsal).* Staff who have attended the education and training session will be encouraged to use this tool and apply this knowledge on their respective wards, and will be advised that their leadership on the local implementation may be an example to other staff who were not in attendance *( 13.1 Identification of self as a model).*
◦ Post education and training session, the RAPID tool will be available on the wards *( 12.5 adding objects to the environment).* Nursing home staff will be prompted to place the RAPID tool in a prominent location (e.g. resident’s care plan) to remind staff to complete it every time a resident exhibits behavioural symptoms *( 7.1 Prompts/cues, 8.3 Habit formation).* Staff will be encouraged to compete the RAPID tool in conjunction with each other (i.e. nurses and healthcare assistants) with input from GPs, family members and residents, where appropriate *( 12.2 Restructuring the social environment).*

•    The three intervention functions directed at GPs will include: **Education, Environmental Restructuring and Persuasion.**

◦ During the academic detailing session, GPs will be provided with written and oral information regarding the risks and benefits of antipsychotics *( 5.1 Information about health consequences)* from a trained academic detailer pharmacist or physician *( 9.1 Credible source).* After presenting the evidence, GPs will be asked to consider antipsychotics as the last resort when dealing with the majority of behavioural symptoms, rather than the first-line treatment *( 13.2 Framing/re-framing)*, and will be encouraged to recommend non-drug alternatives instead of prescribing antipsychotics in these instances *( 8.2 Behaviour substitution).*
◦ As part of the academic detailing session, GPs will be introduced to the RAPID assessment tool. However responsibility for its completion will lie with the nursing home staff. GPs will be prompted by staff to review completed RAPID assessment tools when they come to do their ward round, by having them placed in a prominent place (e.g. care plans) ( *7.1 Prompts/cues, 12.5 Adding objects to the environment*). As above, The RAPID tool will focus GPs‘ attention on identifying and exploring patterns of events and triggers that occur in residents (e.g. repetitive actions, sun-downing, pain) ( *4.2 Information about antecedents*) that may ultimately lead to an inappropriate prescription of an antipsychotic, and to develop non-drug strategies to use in these situations to address these factors ( *1.2 Problem solving*). Nursing home staff will be encouraged to outline a detailed plan of how and when non-drug and/or drug interventions will be utilised in such situations ( *1.4 Action Planning, 1.2 Problem solving*), in conjunction with the GP and others ( *12.2 Restructuring the social environment*).

Another noticeable change was in relation to the RAPID assessment tool that we developed for the study. A Director of Nursing, who acted as a professional stakeholder advisor for this project, piloted this tool in her nursing home of 100 residents and provided oral feedback to the research team. The main feedback was that the tool was too long and should be substantially shorter. This advice helped to shape the final instrument.

Of note, since the development of this intervention, the COVID-19 pandemic has resulted in millions of cases and deaths worldwide ^42^. Given the particular mortality risk that COVID-19 presents to nursing home residents with dementia ^15^, along with the increased risk of infection among health and social care staff ^43^, changes to the format of the delivery of this intervention may be required. Moving from a face-to-face to an online or blended delivery of nursing home staff education, will help to mitigate the risk of infection posed by this intervention. However, care is required to ensure that the identified BCTs for the intervention are not lost in translation.

### 11. Changes to interventions required for subgroups

From our discussions with professional stakeholder groups, it was evident that GPs would only have limited time to avail of any proposed education. Whereas, nursing staff, subject to availability and support from management, may prefer a classroom-based activity over a longer period, and ideally removed from any work commitments. Hence, targeted academic detailing sessions provided on-site, and lasting less than 20 minutes were considered preferable to GPs, while two-day group-based education and training activities, provided off-site, were considered preferable to nursing staff. In light of the COVID-19 pandemic however, the mode of delivery (i.e. face-to-face, online or blended and individual or group-based) may need to change as discussed in item 10.

The importance of a credible source delivering the education and training was an identified BCT ^23^, however some flexibility as to the professional status of the educator/trainer was permissible. Based on the Diffusion of Innovations theory, we theorised that homophilous communication (i.e. between individuals with similar attributes) would be important to incorporate when educating nursing staff (i.e. via a local nurse) on nursing elements of dementia care, because these types of communications tend to be more effective, as people can relate better to a facilitator who is similar in most attributes to them ^40^. However, a certain degree of heterophilous communication (i.e. between individuals with different attributes) is also believed to be important, when introducing new ideas into a setting ^40^. Hence a pharmacist or a physician may be a suitable educator to discuss pharmacological or medical issues relating to antipsychotics. Similarly, for the academic detailing for GPs, the person delivering the intervention should be viewed as knowledgeable on the topic of antipsychotic prescribing and should be trained in academic detailing. Hence, a suitably trained pharmacist or physician may be best placed to deliver the academic detailing component. Ideally, we viewed a combination of professionals with different skills and areas of expertise to be the most appropriate team to deliver the various education and training elements ^38^.

### 12. Uncertainties at the end of the development process

At the conclusion of the intervention development process, we had a good understanding of the rationale for this intervention and the underpinning evidence and theory. However, uncertainties remained regarding the intensity (i.e. the length and frequency of education/training), the uptake necessary for effective implementation, the utility of the intervention materials, the role of family members, and the acceptability and effectiveness of the intervention.

### 13. The developed intervention

Having carefully considered the evidence, the relevant behaviour change and implementation theories, the expert opinion of various stakeholders and our PPI advisory groups, and working within the framework of our five intervention functions and 16 BCTs, we agreed upon the following three components for the RAPID complex intervention (and linked to the relevant implementation strategies from the EPOC taxonomy) ^26^:

1. Education and training sessions with nursing home staff (Educational meetings, Educational materials)
2. Academic detailing with GPs (Educational outreach visits/academic detailing, Educational materials)
3. Introduction of an assessment tool to the nursing home environment (Local opinion leaders, Patient-mediated interventions, Reminders).

The BCT composition of the RAPID complex intervention is described in Box 1. Additionally, we have specified the details of the intervention according to the TIDieR checklist (Extended Data Table 3) ^24, 28^. The RAPID assessment tool developed for this complex intervention is available online (Extended Data File 4) ^28^.

### 14. Open access format

The current publication is open access and materials connected to this publication are freely available online (Extended Data) ^28^.

## Discussion

This paper describes in a transparent and structured manner using the GUIDED checklist ^17^, our intervention development process for the RAPID complex intervention. In this paper, we detail the RAPID complex intervention and explain the decision-making process by describing: the context; the purpose; the target population; the underpinning theory and evidence; the use of existing interventions; our guiding principles; stakeholder involvement; and changes and outstanding uncertainties. We enhanced the GUIDED reporting of the intervention development process by drawing on other frameworks and tools including the TIDieR checklist ^24^; the CICI framework ^25^; the EPOC taxonomy ^26^, the UK Medical Research Council framework ^18^; the Behaviour Change Wheel ^19^; the Theoretical Domains Framework ^20^; and the GRIPP2-SF checklist ^27^.

By outlining the intervention development process in an open and transparent manner, we believe that this will facilitate the scale up, replication, iteration and optimisation of the intervention going forward. We also believe that the approach to intervention development described in this paper could be used as an exemplar for future interventions. Intervention development is an evolving science, and methodological papers like this can help intervention developers to understand the various methods and approaches that can be used. Therefore lessons can be learned and incorporated into future intervention development studies ^17^. We found that the GUIDED checklist complemented other tools and framework and that it provided a useful way of reporting all of these elements in a cohesive manner.

The next step of this project is to report the feasibility study for the RAPID complex intervention, in line with the next phase of the UK Medical Research Council framework ( Figure 1) ^18^. Ultimately, we plan to evaluate the effectiveness of the RAPID complex intervention in a large scale randomised controlled trial (RCT). We believe that transparency in the intervention development and evaluation processes can help in the implementation of evidence-based practice. By presenting effectiveness studies alongside intervention development and feasibility studies, this will enable healthcare professionals and commissioners to understand the context and methods that were used to develop and evaluate the intervention, and so judgements about the quality and relevance of the intervention can be better informed. This detailed information could help inform decisions as to whether an intervention should be implemented in a specific setting or not, or how it might be adapted to a specific setting ^17^.

A key step of the Behaviour Change Wheel is to identify policy categories through which the intervention could be implemented ^19^. The Behaviour Change Wheel guidance outlines seven distinct policy categories that can be considered when developing an intervention (communication/marketing, guidelines, fiscal measures, regulation, legislation, environmental/social planning and service provision) ( Figure 2). We did not make any linkages to policy categories as we did not have access to any policy levers at the time of undertaking the project ^19^. However, since the completion of this project, several of the research team have lead on the development of a National Clinical Guideline on the appropriate prescribing of psychotropic medication for non-cognitive symptoms in people with dementia ^8^. Although conducted separately to the RAPID complex intervention, this National Clinical Guideline has the potential to be a policy vehicle through which this intervention could be implemented nationally.

A particular challenge that we encountered while undertaking this study was combining all the various tools and frameworks along with the perspectives of a broad range of stakeholders. Although we have presented these in a cogent and logical manner in the current study, it required careful consideration by the research team. While some tools and frameworks are reasonably well integrated with one another (i.e., the TIDieR checklist ^24^, the UK Medical Research Council framework ^18^, the Behaviour Change Wheel ^19^, and the Theoretical Domains Framework) ^20^ others did not fit as neatly and so we only used certain domains (i.e. the CICI framework ^25^, and EPOC taxonomy) ^26^ or an abridged version of the tool (i.e. and the GRIPP2-SF checklist) ^27^. Coming to consensus was also challenging at times due to differing perspectives. However, we strived to achieve a balance between the views and preferences of our advisory groups with those of healthcare workers working in the nursing home setting. Hence, while family members were not directly targeted by the developed intervention per se, their involvement was strongly encouraged.

Another limitation of the intervention development process is that it was conducted prior to the onset of the COVID-19 pandemic, as nursing homes across the world are seeing high levels of mortality in residents due to outbreaks of the virus ^44^, and hence some of the assumptions underpinning the intervention may no longer hold true. For example, in the qualitative studies which informed the intervention, a broad range of barriers and facilitators to appropriate antipsychotic prescribing to nursing home residents were identified ^21, 22^. However, none related to the need to ensure compliance with infection prevention and control measures (e.g. hand washing, mask wearing, social distancing, isolation and quarantine) or to alleviate the anxiety that a pandemic potentially evokes among both staff and residents, as these issues did not appear to influence decision-making in the context of antipsychotic prescribing. Concerns have recently been expressed that antipsychotics are potentially being used more, due to a combination of factors including the need for compliance with such measures among residents, to reduce anxiety induced by the pandemic and related measures, and when the usual meaningful and therapeutic activities to prevent or reduce BPSD have been dramatically reduced ^15, 16^. Hence, the reasons for antipsychotic prescribing to nursing home residents may be different now compared to when our research was initially conducted, and so additional research may be required to understand the behavioural determinants of antipsychotic prescribing in the context of COVID-19.

## Conclusion

This paper describes the steps used by the research team to develop a complex intervention targeting antipsychotic prescribing to nursing home residents with dementia in Ireland, according to the GUIDED checklist. We found that the GUIDED checklist provided a useful way of reporting all elements in a cohesive manner and complemented other tools and frameworks. We believe that the approach to intervention development described in this paper could be used as an exemplar for future interventions, and that transparency in the intervention development process can help in the translation of evidence into practice. The next step involves reporting the feasibility study of the developed RAPID complex intervention.

## Data availability

### Underlying data

All data underlying the results are available as part of the article and no additional source data are required.

### Extended data

Figshare: Extended Data: Developing a complex intervention targeting antipsychotic prescribing to nursing home residents with dementia. https://doi.org/10.6084/m9.figshare.13668929.v1 ^28^.

This project contains the following extended data:

- GUIDED – a guideline for reporting intervention development studies.
- RAPID assessment tool.

### Reporting guidelines

In addition to the current paper which details the development process in accordance with the GUIDED checklist, the following reporting guidelines were adhered to:

GRIPP2-SF Checklist for ‘Developing a complex intervention targeting antipsychotic prescribing to nursing home residents with dementia’. https://doi.org/10.6084/m9.figshare.13668929.v1 ^28^.

TIDieR Checklist for’ Developing a complex intervention targeting antipsychotic prescribing to nursing home residents with dementia’. https://doi.org/10.6084/m9.figshare.13668929.v1 ^28^.

Data are available under the terms of the Creative Commons Attribution 4.0 International license (CC-BY 4.0).
