# Supplementary material for: Developing a complex intervention targeting antipsychotic prescribing to nursing home residents with dementia
Source: HRB Open Res. 2021 Feb 19;4:23. [Version 1] doi: 10.12688/hrbopenres.13228.1 (PMC8185579; doi:10.12688/hrbopenres.13228.1)
Supplement: Peer review history

# Peer review: Reviewer response for version 1

**DOI:** 10.21956/hrbopenres.14386.r29441

**Published:** 2021-06-07

**Authors:** Sion Scott

**Affiliations:** 1 School of Pharmacy, University of East Anglia, Norwich, UK

Thank you for inviting me to review this manuscript. This is an excellent, transparent overview of the development of a theory and evidence based intervention, and I commend the author’s for producing this. We need to see more publications like this so that we can understand how interventions were developed, and how they are hypothesised to work. This also serves as a useful resource for early career researchers to see how it is done!

Is the work clearly and accurately presented and does it cite the current literature?

Yes

If applicable, is the statistical analysis and its interpretation appropriate?

Not applicable

Are all the source data underlying the results available to ensure full reproducibility?

Yes

Is the study design appropriate and is the work technically sound?

Yes

Are the conclusions drawn adequately supported by the results?

Yes

Are sufficient details of methods and analysis provided to allow replication by others?

Yes

Reviewer Expertise:

Behavioural scientist - PhD in deprescribing intervention development.

I confirm that I have read this submission and believe that I have an appropriate level of expertise to confirm that it is of an acceptable scientific standard.

# Peer review: Reviewer response for version 1

**DOI:** 10.21956/hrbopenres.14386.r29353

**Published:** 2021-06-07

**Authors:** Carole Parsons

**Affiliations:** 1 School of Pharmacy, Queen's University Belfast, Belfast, UK

This is an extremely well written article outlining the development of a complex intervention to improve requesting and prescribing of antipsychotic medications for nursing home residents with dementia. The intervention development process was well documented and drew on a number of appropriate frameworks and tools. The process was clearly described and the standard of English was consistently high. My preference would be to change "prescribing to" to "prescribing for" nursing home residents with dementia, and changing "was comprised of" to "comprising"; however these are very minor.

Is the work clearly and accurately presented and does it cite the current literature?

Yes

If applicable, is the statistical analysis and its interpretation appropriate?

Not applicable

Are all the source data underlying the results available to ensure full reproducibility?

Yes

Is the study design appropriate and is the work technically sound?

Yes

Are the conclusions drawn adequately supported by the results?

Yes

Are sufficient details of methods and analysis provided to allow replication by others?

Yes

Reviewer Expertise:

Medicines optimisation and prescribing for older people with advanced illness or receiving palliative care.

I confirm that I have read this submission and believe that I have an appropriate level of expertise to confirm that it is of an acceptable scientific standard.
